# *Muntingia calabura* Leaves Mediated Green Synthesis of CuO Nanorods: Exploiting Phytochemicals for Unique Morphology

**DOI:** 10.3390/ma14216379

**Published:** 2021-10-25

**Authors:** Vidhya Selvanathan, Mohammod Aminuzzaman, Lai-Hock Tey, Syaza Amira Razali, Khaled Althubeiti, Hend Ibraheem Alkhammash, Samar Kumar Guha, Sayaka Ogawa, Akira Watanabe, Md. Shahiduzzaman, Md. Akhtaruzzaman

**Affiliations:** 1 Solar Energy Research Institute (SERI), Universiti Kebangsaan Malaysia (UKM), Bangi 43600, Malaysia; vidhya@ukm.edu.my (V.S.); mirarazali@gmail.com (S.A.R.); 2 Department of Chemical Science, Faculty of Science, Universiti Tunku Abdul Rahman (UTAR), Perak Campus, Jalan Universiti, Bandar Barat, Kampar 31900, Malaysia; teylh@utar.edu.my; 3 Centre for Photonics and Advanced Materials Research (CPAMR), Universiti Tunku Abdul Rahman (UTAR), Jalan Sungai Long, Bandar Sungai Long, Kajang 43000, Malaysia; 4 Department of Chemistry, College of Science, Taif University, P.O. Box 11099, Taif 21944, Saudi Arabia; k.althubeiti@tu.edu.sa; 5 Department of Electrical Engineering, College of Engineering, Taif University, Taif 21944, Saudi Arabia; Khamash.h@tu.edu.sa; 6 Department of Arts and Sciences, Faculty of Engineering, Ahsanullah University of Science and Technology, 141-142, Love Road, Tejgaon I/A, Dhaka 1208, Bangladesh; skguha.as@aust.edu; 7 Institute of Multidisciplinary Research for Advanced Materials (IMRAM), Tohoku University, Sendai 980-8577, Japan; sayaka.ogawa.a1@tohoku.ac.jp (S.O.); akira.watanabe.c6@tohoku.ac.jp (A.W.); 8 Nanomaterials Research Institute (NanoMaRi), Kanazawa University, Kakuma, Kanazawa 920-1192, Japan; shahiduzzaman@se.kanazawa-u.ac.jp

**Keywords:** copper oxide, nanorods, green synthesis, *Muntingia calabura*

## Abstract

In this study, phytochemical assisted nanoparticle synthesis was performed using *Muntingia calabura* leaf extracts to produce copper oxide nanoparticles (CuO NPs) with interesting morphology. Scanning electron microscope (SEM) and transmission electron microscope (TEM) analysis of the biosynthesized CuO NPs reveal formation of distinct, homogeneous, and uniform sized CuO nanorods structure with thickness and length of around 23 nm and 79 nm, respectively. Based on Fourier-transform infrared (FTIR) analysis, the unique combinations of secondary metabolites such as flavonoid and polyphenols in the plant extract are deduced to be effective capping agents to produce nanoparticles with unique morphologies similar to conventional chemical synthesis. X-ray diffraction (XRD) analysis verified the monoclinical, crystalline structure of the CuO NPs. The phase purity and chemical identity of the product was consolidated via X-Ray photoelectron spectroscopy (XPS) and Raman spectroscopic data which indicate the formation of a single phase CuO without the presence of other impurities. The direct and indirect optical band gap energies of the CuO nanorods were recorded to be 3.65 eV and 1.42 eV.

## 1. Introduction

Copper oxide nanoparticles (NPs) are the multifunctional members of the family of copper compounds, with unique properties compared to its macroscopic counterparts. Copper oxide in the stoichiometric form of CuO, also known as cupric oxide, is a p-type semiconductor with band gap of 1.7 eV. Due to their high surface area to volume ratio, CuO NPs are widely used in sensors, energy storage devices, antimicrobial activity, environmental remediation, and catalytic applications [1,2,3,4]. To date, several experimental methods have been utilized to synthesize CuO NPs, including sol-gel process [5], electrochemical approach [6], sonochemistry [7], and microwave irradiation [8]. While the conventional wet chemistry method was very effective in producing homogeneously sized CuO NPs, the environmental hazards posed by the usage of certain chemical species remained a major concern [9,10]. This dilemma paved the way for novel attempts to synthesize CuO NPs using plant extracts as the more sustainable method [11,12,13]. The biosynthesis of different metal oxide nanostructures including nickel oxide [14], cobalt oxide [15], iron oxide [16], and zinc oxide [17] have been reported using various parts of different plant species [18]. To date, a variety of phytochemicals containing plant extracts such as *Cordia sebestena* flower [19], *Rheum palmatum* L. root [20], *Euphorbia pulcherrima* flower [21], and *Caesalpinia bonducella* seed [22] have been reported for successful synthesis of CuO NPs.

While the structural integrity and elemental purity of such green-synthesized CuO NPs have been on par with those synthesized via traditional chemical methods, the morphological properties of the nanomaterial still have room for improvement [23]. In contrast to interesting morphologies, including nanorods, nanowires, nanosheets, and nanoflowers that are reported for chemically synthesized CuO NPs [24,25], green synthesis most often yields spherical or quasi-spherical nanoparticles that are agglomerated into a cluster. This might restrict the application of green synthesized CuO NPs as particle size and shape of nanoparticles govern their physicochemical properties, which in turn dictates their functionality. For instance, Ajibade et al. compared the photocatalytic properties of platinum disulfide nanoparticles in different morphologies such as bead-shape, quasi-spherical and spherical [26]. It was evident that the shape of the nanoparticles affected effective surface area, porosity, and band gap of the material which in turn influenced its efficiency in photocatalytic degradation of dye molecules. In this regard, the choice of plant extract used during the synthesis process plays a crucial role in the mechanism of ion reduction, capping, and stabilization of CuO NPs, ultimately influencing the morphology of the nanostructure.

Hence, in this study, we have attempted to use *Muntingia calabura* leaf extracts for the synthesis of CuO nanorods. *M. calabura* is synonymously known as “Jamaican cherry” throughout the world, and in Malaysia it is usually cultivated as roadside trees [27]. The leaves of this tropical plant species have been traditionally used as tranquillizer, headache remedy, and tonic in several cultures. Recent studies, aimed to determine the bioactive compounds in *M. calabura*, reveal that the leaves are rich in glycosides, tannins, flavonoids, and terpenoids [28,29,30,31]. This unique cocktail of phytochemicals in the *M. calabura* leaves extract are exploited as an ideal medium for synthesis of CuO NPs with unique morphology.

## 2. Materials and Methods

### 2.1. Materials

Fresh *Muntingia calabura* leaves were collected from roadside trees in Kampar, Malaysia. Copper (II) nitrate trihydrate [Cu(NO_3_)_2_.3H_2_O] was purchased from Quality Reagent Chemical, QRëC, New Zealand, and used as purchased.

### 2.2. Preparation of Plant Extract

Freshly plucked *M. calabura* leaves were washed with tap water and allowed to air dry at room temperature for 5 days. Once dried, the leaves were ground to fine powder. In the preliminary step, CuO NPs were synthesized by using different amounts (0.5 g, 1 g, 2 g, and 5 g) of *M. calabura* leaves powder suspended in 100 mL deionized water, followed by heating at 80 °C for 20 min [32,33,34]. However, the best formation of nanoparticles was achieved with the concentration of 2 g of *M. calabura* leaves in 100 mL water. The light brown solution was then filtered to remove solid particles and stored in a refrigerator.

### 2.3. Green Synthesis of CuO Nanoparticles

**Figure 1:**
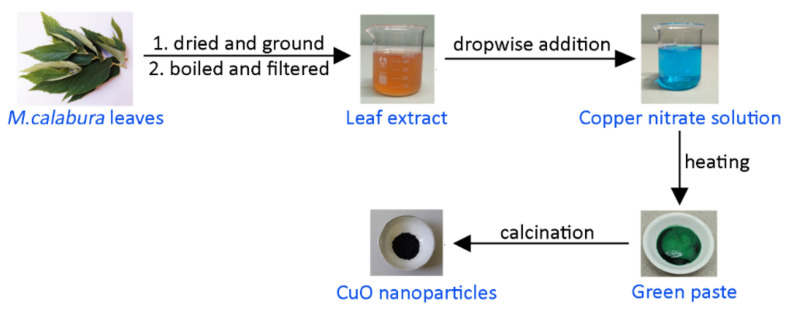
Experimental procedure for green synthesis of CuO nanoparticles.

For the synthesis of CuO NPs, 50 mL solution of 0.5 M copper (II) nitrate trihydrate was prepared. We added 10 mL of the *M. calabura* leaves extract dropwise to the copper precursor solution and the mixture was stirred at 80 °C for 4 h. This resulted in a greenish paste which was allowed to cool to room temperature. The paste was calcined at 400 °C for 2 h, producing fine, black CuO powder. Figure 1 summarizes the synthesis of natural extract mediated CuO NPs.

### 2.4. Characterization of CuO Nanoparticles

The crystal properties of the sample were characterized from 10 to 80° in 2*θ* by an X-ray diffractometer with CuK*α* radiation (Shimadzu XRD 6000, Kyoto, Japan). Morphological characteristics were characterized by FESEM (JEOL JSM-6701F combined with EDX, Tokyo, Japan) and high resolution transmission electron microscope (HRTEM) (JEOL JEM 3010). UV–Vis absorption spectra were recorded by a UV–visible spectrophotometer (Perkin Elmer Lambda 35, (Waltham, MA, USA). The FTIR spectra of biosynthesized CuO NPs were recorded by KBr pellet method using FTIR spectrophotometer (Perkin Elmer RX1). The X-ray photoelectron spectra were obtained using Perkin Elmer PHI5600 (ULVAC-PHI, Inc.,Waltham, MA, USA). A micro-Raman spectrometer equipped with an optical microscope (Olympus BX51, Tokyo, Japan), a CW 532 nm DPSS laser, a Peltier-cooled CCD camera (DV401, Andor Technology, Belfast, UK) and a monochromator (MS257, Oriel Instruments Co., Stratford, CT, USA) were used to measure the Raman spectra.

## 3. Results and Discussions

### 3.1. Structural Analysis

**Figure 2:**
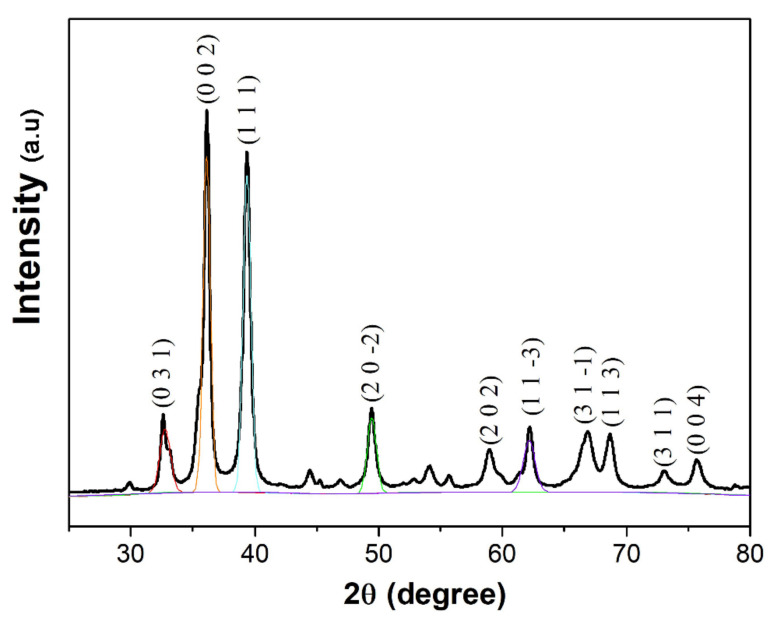
X-ray diffractogram of *M. calabura* derived CuO nanoparticles.

The X-ray diffractogram of *M. calabura* mediated CuO nanoparticles depict sharp, intense peaks, which verifies the crystalline nature of the nanostructures. As shown in Figure 2, major diffraction peaks were evident at 2*θ* values of 32.51°, 36.32°, 39.20°, 49.42°, and 62.26° which are attributed to (0 3 1), (0 0 2), (1 1 1), (2 0 −2), and (1 1 −3) planes, respectively. Additionally, the minor peaks detected at 58.87°, 66.90°, 68.70°, 73.05°, and 75.68° can be assigned to the crystal planes of (2 0 2), (3 1 −1), (1 1 3), (3 1 1), and (0 0 4), respectively. The peaks detected in this study coincide with previously reported literature on CuO NPs [35,36,37] and is in agreement with International Centre for Diffraction Data (ICDD): Entry number-00-045-0937. Based on the XRD peak positions, it can be concluded that the CuO NPs synthesized via the phytochemical method has a monoclinic phase with lattice parameters of a = 4.6853 Å, b = 3.4257 Å, c = 5.1303 Å, and β = 99.549 Å The distinct, well-defined XRD peaks without any impurity peaks is a further testament to the purity of CuO NPs obtained using the reported method.

To gain an insight on the average crystallite size (*D*) of the CuO NPs, the XRD peaks were analysed using the Debye-Scherrer equation, which states:

(1) $$D=\frac{k\lambda }{\beta cos\theta \;},$$

where *k* is the shape constant (taken to be 0.9), *λ* is the wavelength of the X-ray radiation (in nm), *β* is the full width half maximum of the peak (in radians), and *θ* is the diffraction angle (in degrees). Table 1 shows the average crystallite size based on the prominent XRD peaks which ranges between 12 to 20 nm. It is crucial to note that the crystallize size of non-spherical structures calculated using this method may have deviations due to the effect of shape factor value used in Debye–Scherrer’s formula. Besides that, occurrence of non-single crystal, heterogeneous crystal strain, and instrumental effects may also deviate the calculated value from actual ones.

**Figure 3:**
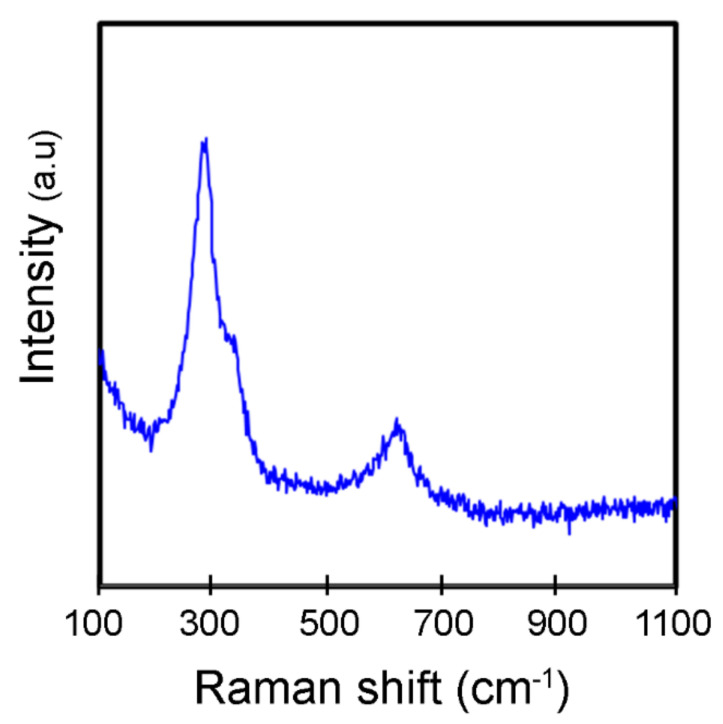
Raman spectrum of *M. calabura* derived CuO nanoparticles.

The monoclinic CuO nanocrystal structure and phase purity were further validated based on the Raman spectrum of the product as shown in Figure 3. As the space group of CuO is C^6^_2h_, the zone centre Raman active normal modes of CuO are ΓRA = 4A_u_ + 5B_u_ + A_g_ + 2B_g_. Between these vibration modes, there are three Raman active modes, namely one A_g_ and two B_g_ [38]. The Raman spectrum of the CuO NPs clearly shows peaks at 288, 324, and 621 cm^−1^, which corresponds to the typical modes of A_g_ and two B_g_, respectively. The wavenumbers detected in this study are found to be at lower values compared to Raman spectra of CuO nanostructures prepared by microwave irradiation method in previous literature (296, 346, and 631 cm^−1^) [38]. This could be an impact of smaller grain size of the CuO NPs, which is reported to shift the Raman bands to smaller wavenumbers [39].

**Figure 4:**
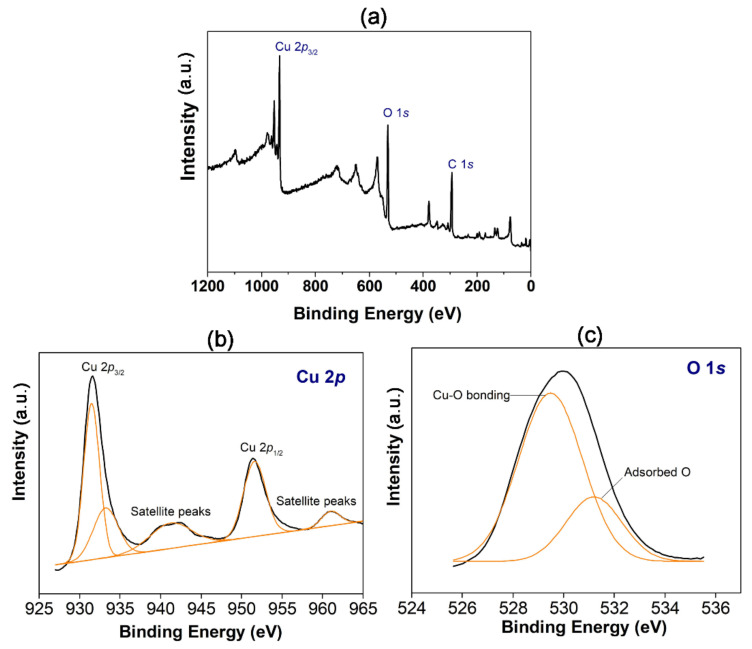
(**a**) XPS survey spectra of *M. calabura* derived CuO nanoparticles, (**b**) high-resolution Cu 2*p* peaks, and (**c**) high-resolution O 1*s* peaks.

To comprehend the chemical composition of the green synthesized CuO NPs, the sample was subjected to XPS analysis and the survey scan of XPS spectrum depicts peaks corresponding to C 1*s*, O 1*s*, and Cu 2*p* (Figure 4a). Figure 4b depicts the high resolution Cu 2*p* spectrum with binding energies of 931.6 and 951.4 eV that are indicative of Cu 2*p*_3/2_ and Cu 2*p*_1/2_, respectively, which correspond to Cu^2+^ state of valency. The gap between both the peaks is around 20 eV, which coincides with standard CuO XPS spectrum. The Cu 2*p*_3/2_ shakeup satellite peak appears at about 941.5 eV and the satellite peak of Cu 2*p*_1/2_ lies at about 961.5 eV. Both these values are consistent with other CuO NPs systems [40,41]. The occurrence of shakeup satellite structures for Cu 2*p* spectrum eliminates the possibility of any Cu_2_O phase present in the product. The O 1*s* spectrum (Figure 4c) of particles exhibit the binding energy allocated at 529.46 eV which corresponds to O 1*s* of Cu-O bonding. In addition, the Gaussian fitting between 526–536 eV region resolves another peak at 531.32 eV that is attributed to presence of surface adsorbed oxygen. Overall, the XPS analysis of green synthesized CuO NPs confirms that the product is in a single phase without presence of other impurities.

### 3.2. Optical Analysis

**Figure 5:**
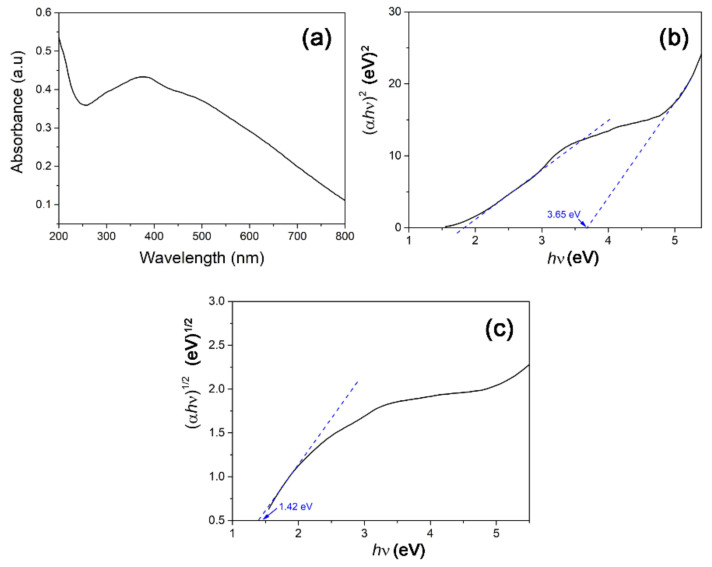
(**a**) UV–Vis absorption spectrum of *M. calabura* derived CuO nanoparticles and the corresponding Tauc plot for (**b**) direct and (**c**) indirect band gap.

The UV–Vis absorption spectrum of the CuO NPs was analysed by forming a 0.1 wt% dispersion of the product using deionized water. The UV–Vis absorption spectrum (Figure 5a) illustrates a rather broad absorption which peaks at 387 nm and is most probably attributed to surface plasmon resonance (SPR) due to semiconductor excitation of CuO [42]. Using the Tauc plot approach, the band gap energy (*E_g_*) of the CuO NPs was derived with the following equation:

(2) $$\alpha h\nu =A{\left(h\nu -E_{g}\right)}^{n},$$

where *h* is incident photon energy, *n* is the exponent factor that governs the electronic transition (*n* = 1/2 for direct band and *n* = 2 for indirect band), and *α* is the absorption coefficient. Figure 5b depicts the extrapolation of the Tauc plot with the direct optical band gap energy of the CuO NPs attaining 3.65 eV. The direct band gap recorded in this study is similar to CuO nanoellipsoids synthesized by Boltaev et al. using the laser ablation method [43]. The higher bandgap of the synthesized nanorods compared to bulk CuO is attributed to the quantum size effect. When particle size approaches the nanoscale dimension, the overlapping of adjacent energy levels minimizes and subsequently the width of energy band widens [44]. As the optical properties of the CuO nanorods also indicate the presence of indirect band gap, the Tauc plot derivative for indirect optical bandgap calculation is depicted in Figure 5c. The nanorods show an indirect band gap of 1.42 eV, which coincides with the reported literature value for monoclinic cupric oxide structure [45].

### 3.3. Morphological Analysis

**Figure 6:**
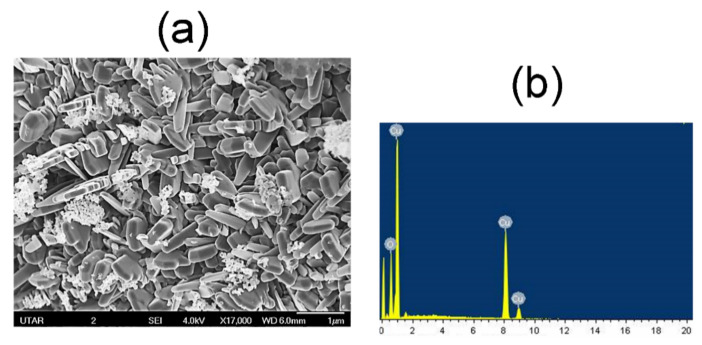
(**a**) FESEM images and (**b**) EDX spectrum of *M. calabura* derived CuO nanoparticles.

The structural properties of CuO NPs were investigated using FESEM analysis, as shown in Figure 6a. The nanoparticles seemingly displayed a rod-like morphology with uniformity in terms of size and particle distribution. In fact, the nanorods appear as distinct structures without much agglomeration, as opposed to agglomerated clusters often observed in most phytochemical assisted CuO NP synthesis. Figure 6b shows the elemental compositions of the sample gauged using EDX analysis, which reveal copper and oxygen as the sole elements present. 

**Figure 7:**
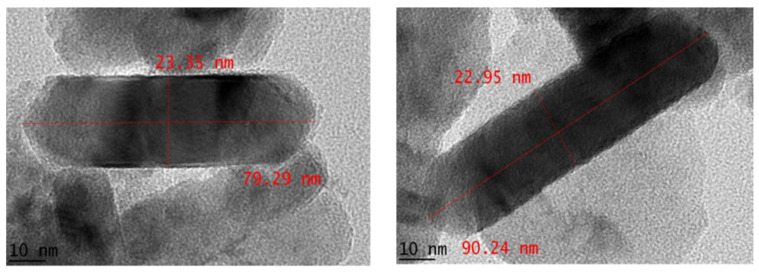
TEM images of *M. calabura* derived CuO nanoparticles.

To elucidate the nanoparticle size and morphology, TEM analysis was performed. Figure 7 shows that the green synthesized CuO nanostructures are clearly in rod shape with thickness around 23 nm and length between 79 to 90 nm. The particle size demonstrated in morphological analysis was bigger than the crystallite size estimated from XRD analysis, hence indicating that the single CuO particle is composed of few crystallites. Table 2 compares the morphological properties of previously reported CuO NP synthesis using phytochemicals. It is evident that the nanoparticle morphology obtained in this study is novel compared to the other systems, and this is an effect of the unique combinations of biomolecules present in the *M. calabura* leaves extract. Particularly, synthesis of CuO nanorods is usually achieved via addition of strong bases, chemical surfactants, or capping agents. In this work, the CuO nanorods can be achieved by simply using the *M. calabura* leaves extract in place of these chemicals. In fact, no organic solvent is used for extraction of phytochemicals from the plant. It is interesting to note that a similar nanostructure can be obtained by employing a green extract, allowing the synthesis process to be cheaper and more sustainable.

### 3.4. Phytochemical Constituents

**Figure 8:**
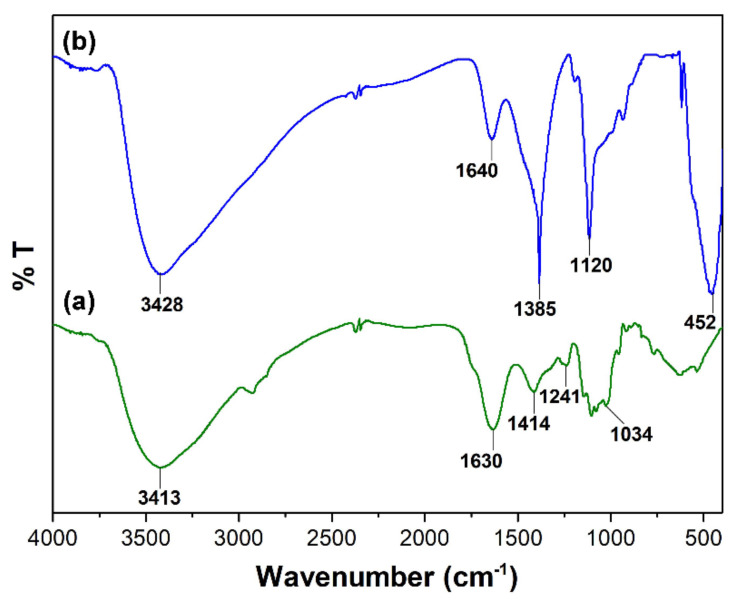
FTIR spectra of (**a**) *M. calabura* extract and (**b**) CuO nanoparticles.

The *M. calabura* leaves extract was characterized using FTIR analysis to explore the presence of active groups in the plant extract that facilitates the formation of CuO nanorods. Figure 8a shows the FTIR spectrum of the *M. calabura* extract with prominent absorption bands at 3413 cm^−1^ and 1630 cm^−1^, corresponding to the O-H bond of the polyphenols and C=O stretch of the flavonoids. The bands at 1414 cm^−1^ and 1034 cm^−1^ originate from –COO carboxylic acid and C-N amine stretch, respectively [19]. Absorption bands due to the carbohydrate components in the leaves extract are visible between 1000 to 1100 cm^−1^. The functional group identities of the *M. calabura* extract indicates the presence of useful secondary metabolites such as glycosides, tannins, flavonoids, and terpenoids, as reported in earlier studies. The traces of these biological compounds are detectible in the FTIR spectrum of the green synthesized CuO NPs (Figure 8b). In particular, the strong absorption bands at 3428 cm^−1^ and 1640 cm^−1^ in the nanostructures indicate that the phenolic group and flavonoids served as encapsulating and stabilizing agents during the formation of CuO nanoparticles [51]. The intense peak at 452 cm^−1^ is recognized to be the characteristic peaks of CuO. Previously, Senthilkumar et al. studied a possible mechanism for the synthesis of ZnO NPs using Tectona grandis L. leaf extract [52]. They suggested that phenols and flavonoids in the aqueous leaf extract bind the surface of zinc in precursor to activate the formation of nanoparticles. The FTIR analysis of CuO NPs in this case show comparable results to their observation.

**Figure 9:**
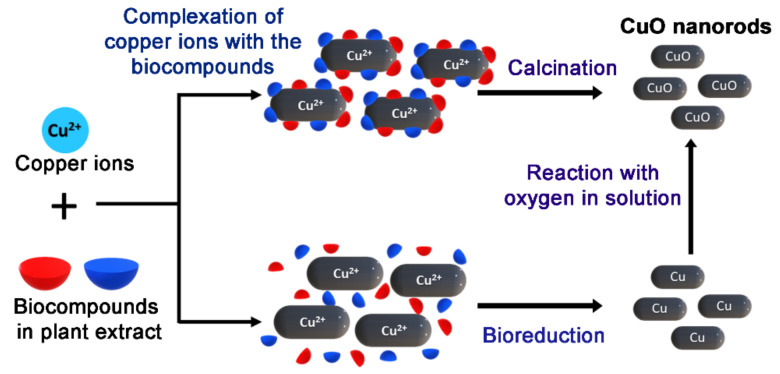
Possible reaction mechanism for *M. calabura* mediated CuO nanoparticles synthesis.

Figure 9 illustrates the possible mechanisms adapted for the formation of CuO nanorods with the aid of biomolecules in *M. calabura* extract. To date, two different approaches have been suggested in the literature to comprehend the plausible mechanism route of the biosynthesis of metal oxide nanoparticles using phytochemicals. In the first approach, the active ingredients in the plant extract, such as flavonoids and phenols, chelate with the precursor copper ions to form coordinated complexes [17]. These intermediate complexes would then thermally degrade upon calcination and form copper oxide nanorods. Several studies reported earlier supports the chelation theory for formation of metal oxide nanoparticles [53,54]. For instance, Matinise et al. (2017) proposed that the antioxidants in Moringa oleiferea leaves chelate with the zinc (II) ions, leading to formation of zinc complexes, which is later converted to zinc oxide nanoparticles after heat treatment [55]. They further corroborated this mechanism by observing FTIR absorption bands characteristic to the bioactive compounds in the plant extract being present in the green synthesized zinc oxide nanoparticles. Alternatively, Singh et al. proposed a bioreduction mechanism for the green synthesis of zinc oxide quantum dots using the mixture of Eclipta alba leaf extract and zinc acetate [56]. In this mechanism, the first step involves the reduction in metal ions to zero-valent states using the bioreducing agents present in the plant extract. The reduced metal atoms are then converted to ZnO as a result of the reaction with the dissolved oxygen (O_2_) content in the reaction mixture. A similar mechanism has been suggested in several other studies, adopting green synthesis of metal oxide nanoparticles [57,58]. Interestingly, in the bioreduction mechanism, it is also proposed that the phytochemicals in the extract also aid particle stabilization by hindering agglomeration of the formed nanoparticles.

## 4. Conclusions

The potential application of *M. calabura* leaf extracts as reducing and capping agents for the synthesis of copper oxide nanoparticles (CuO NPs) is explored for the first time. The green synthesis method was successful in producing highly crystalline, pure CuO nanostructures as confirmed by the presence of characteristic peaks in an XRD analysis. Based on XPS and Raman spectrum, the single phase properties and structural integrity of the product were verified. In contrast to the typical spherical nanoparticle clusters observed in previously reported green synthesized CuO NPs, morphological properties of the product in this study depict the formation of well defined, non-agglomerated CuO nanorods with an average thickness of 23 nm and length between 79 to 90 nm. Based on FTIR analysis, it is deduced that the presence of secondary metabolites, particularly flavonoids and phenols in the *M. calabura* leaf extracts, play an important role in directing and stabilizing the unique rod-like morphology of the CuO NPs.

## Figures and Tables

**Table 1:** Particle size distribution based on Debye–Scherrer’s equation.

2*θ*	Miller Index	FWHM (°)	*β* (Radians)	*D* (nm)
32.51	(0 3 1)	0.71	0.0124	11.65
36.32	(0 0 2)	0.41	0.0072	20.38
39.20	(1 1 1)	0.57	0.0099	14.80
49.42	(2 0 −2)	0.63	0.0110	13.88
62.26	(1 1 −3)	0.75	0.0131	12.36

**Table 2:** Comparison of morphological properties of CuO NPs derived from *M. calabura* leaves with recent literature.

Source of Phytochemical	Smallest Particle Size (nm)	Shape	References
*Madhuca longifolia* seeds	30	Quasi-spherical	[46]
*Carica papaya* peel	85	Irregular	[47]
*Anthemis nobilis* flowers	61	Irregular	[48]
*Musa acuminata* peel	50	Spherical	[49]
*Psidium guajava* leaves	33	Spherical	[50]
*Caesalpinia bonducella* seed	13	Rice shaped	[22]
*Muntingia calabura* leaves	23	Rod shaped	This work

## Data Availability

The data presented in this study are available upon request from the corresponding author.
